# Supplementary material for: Oral Microbiota in Children and Adolescents with Type 1 Diabetes Mellitus: Novel Insights into the Pathogenesis of Dental and Periodontal Disease
Source: Microorganisms. 2023 Mar 6;11(3):668. doi: 10.3390/microorganisms11030668 (PMC10059713; doi:10.3390/microorganisms11030668)
Supplement: Supplementary file 1 [file microorganisms-11-00668-s001.zip › microorganisms-2225332-supplementary.pdf]

Supplementary Table S1. Primers and PCR conditions used in the study.

Type	Target	Sequence	Reference	PCR type	Annealing
F	16s rRNA	CGT GCC AGC AGC CGC GGT AAT ACG	Garcia et al., 1998 (1)	mPCR	70°C
R	PI	TCC GCA TAC GTT GCG TGC ACT CAA G			
R	PG	TAC ATA GAA GCC CCG AAG GAA GAC G			
R	AG	CTT TGC ACA TCA GCG TCA GTA CAT CCC CAA GG			
F	TD	GCA AGA CTT GTA GCG GTA GT	Pardo et al., 2021 (2)	mPCR	60°C
R	TD	GAT GCC TAT TTG CGG GCT TG			
F	TF	CGG TGG TCT CCA ATC TCA CC			
R	TF	GCC CTC AAC ACA CGA CAC TT			
F	AN	GGA ATG ATG GCG TGA ATG GC			
R	AN	CCG ATC CCG TGA GTA CAT GG			
F	AC	GGC KTG CGG TGG GTA CGGG C	Xia et al., 2003 (3)	sPCR	60°C
R	AC	GGC TTT AAG GGA TTC GCT CCR CCT CAC			
F	VE	GTA ACA AAG GTG TCG TTT CTC G	Mashima et al., 2016 (4)	sPCR	60°C
R	VE	GCA CCR TCA AAT ACA GGT GTA GC			
F	SM	GGCACCACAACATTGGGAAGCTCAGTT	Nakano et al., 2006 (5)	sPCR	70°C
R	SM	GGAATGGCCGCTAAGTCAACAGGAT			
F	LC	TGGAAACAGRTGCTAATACCG	Byun et al., 2004 (6)	sPCR	62°C
R	LC	GTCCATTGTGGAAGATTCCC			

Abbreviations: F = forward primer; R = reverse primer; mPCR = multiple PCR; sPCR = single PCR; PG = *P. gingivalis*; PI = *P. intermedia*; AG = *A. actinomycetemcomitans*; AN = *A. naeslundii*; TF = *T. forsythia*; TD = *T. denticola*; AC = *Actinomyces* spp.; VE = *Veillonella* spp.; SM = *S. mutans,* LC = *Lactobacillus spp.*

1. García L, Tercero JC, Legido B, Ramos JA, Alemany J, Sanz M. Rapid detection of Actinobacillus actinomycetem-comitans, Prevotella intermedia and Porphyromonas gingivalis by multiplex PCR. J Periodontal Res 1998, 33, 59-64. doi: 10.1111/j.16000765.1998.tb02292.x.
2. Pardo A, Signoriello A, Signoretto C, et al. Detection of Periodontal Pathogens in Oral Samples and Cardiac Specimens in Patients Undergoing Aortic Valve Replacement: A Pilot Study. J Clin Med. 2021;10(17):3874. Published 2021 Aug 28. doi:10.3390/jcm10173874.
3. Xia T, Baumgartner JC. Occurrence of Actinomyces in Infections of Endodontic Origin. J. Endod. 2003, 29, 549–552, doi:10.1097/00004770-200309000-00001.
4. Mashima I, Theodorea CF, Thaweboon B, Thaweboon S, Nakazawa F. Identification of Veillonella species in the tongue biofilm by using a novel one-step polymerase chain reaction method. PLoS One 2016;11(06):e0157516
5. Nakano K, Inaba H, Nomura R, Nemoto H, Takeda M, Yoshioka H, Matsue H, Takahashi T, Taniguchi K, Amano A, et al. Detection of cariogenic Streptococcus mutans in extirpated heart valve and atheromatous plaque specimens. J. Clin. Microbiol. 2006, 44, 3313–3317, doi:10.1128/JCM.00377-06.
6. Byun R, Nadkarni MA, Chhour KL, Martin FE, Jacques NA, Hunter N. Quantitative analysis of diverse Lactobacillus species present in advanced dental caries. J Clin Microbiol. 2004 Jul;42(7):3128-36. doi: 10.1128/JCM.42.7.3128-3136.2004. PMID: 15243071; PMCID: PMC446321.

Supplementary Table S2. Main clinical, biochemical and microbial characteristic of children and adolescents with type 1 diabetes according to glucose management indicator (GMI, cut-off=7.5%)

		All (n=83)	GMI≤7.5% (n=50)	GMI>7.5% (n=33)	p value
**Gender**	**Female (%)**	33 (39.8)	21 (42.0)	12 (36.4)	0.608
	**Male (%)**	50 (60.2)	29 (58.0)	21 (63.6)	
**Age (years)**		12.49±2.18	12.56±2.13	12.37±2.27	0.645
**Diabetes duration (years)**		6.05±2.88	6.13±2.80	5.92±3.04	0.659
**BMI (kg/m²)**		20.08±2.97	19.89±2.65	20.37±3.43	0.506
**Total Insulin (U/kg/die)**		0.87±0.25	0.87±0.23	0.87±0.28	0.823
**Basal Insulin (U/kg/die)**		0.43±0.15	0.44±0.16	0.42±0.14	0.915
**Prandial Insulin (U/kg/die)**		0.40 [0.29-0.55]	0.38 [0.28-0.56]	0.41 [0.34-0.55]	0.539
**Mean glycemia (sensor)**		171.5±30.4	153.0±15.1	199.3±26.1	**>0.001**
**Blood agar, n=82 (CFU × 10⁸/mL)**		10.9±45.5	6.57±5.14	17.9±73.6	0.132
**Sabouraud agar, n=81 (CFU × 10⁴/mL)**		0.38±1.65	0.31±1.48	0.49±1.90	0.548
**Mannitol Salt agar, n=82 (CFU × 10⁸/mL)**		0.12±0.92	0.17±1.15	0.04±0.19	**0.041**
**Mitis Salivarius agar, n=82 (CFU × 10⁷/mL)**		10.3±22.1	10.2±24.0	10.4±18.9	0.474
**MSB, n=82 (CFU × 10⁸/mL)**		0.13±0.33	0.13±0.32	0.13±0.36	0.392
***Veillonella* spp, n(%)**		77 (92.8)	46 (92.0)	31 (93.9)	0.738
***Actinomyces* spp, n(%)**		83 (100)	50 (100)	33 (100)	1
***Actinomyces naeslundii,* n(%)**		39 (47)	24 (48.0)	15 (45.5)	0.820
***Treponema denticola*, n(%)**		30 (36)	19 (38.0)	11 (33.3)	0.665
***A. actinomycetemcomitans*, n(%)**		83 (100)	50 (100)	33 (100)	1
***Prevotella intermedia*, n(%)**		83 (100)	50 (100)	33 (100)	1
***Porphyromonas gingivalis*, n(%)**		6 (7.3)	3 (6.0)	3 (9.0)	0.595
***Tannerella forsythia*, n(%)**		27 (32.5)	13 (26.0)	14 (42.4)	0.118
***Streptococcus mutans,* n(%)**		39 (47)	22 (44.0)	17 (51.5)	0.052
***Lactobacillus* spp, n(%)**		83 (100)	50 (100)	33 (100)	1
***Veillonella* spp + *Streptococcus mutans.*, n(%)**		37 (44.6)	21 (42.0)	16 (48.5)	0.056
***T. forsythia + T. denticola + P. gingivalis* (at least 2 of them), n(%)**		14 (16.9)	6 (12.0)	8 (24.2)	0.124

Sample sire, n=83, unless otherwise indicated. Data are expressed as means ± SD, medians and interquartile range [IQR] or proportions. Differences between the two groups of individuals were tested by the unpaired Student's for normally distributed variables, the Mann–Whitney U-test for non-normally distributed variables or the chi-squared test for categorical variables, respectively. *Abbreviations*: BMI, Body Mass Index; CFU, Colony Forming Unit; MSB, Mitis Salivarius sucrose Bacitracin.

Supplementary Table S3. Main clinical, biochemical and microbial characteristic of children and adolescents with type 1 diabetes according to percentage of time in target range (TIR, cut-off=70%)

		All (n=87)	TIR≥70% (n=22)	TIR<70% (n=65)	**p value**
**Gender**	**Female (%)**	33 (37.9)	9 (40.9)	24 (36.9)	0.739
	**Male (%)**	54 (62.1)	13 (59.1)	41 (63.1)	
**Age (years)**		12.54±2.17	12.87±2.25	12.43±2.15	0.414
**Diabetes duration (years)**		6.13±2.92	6.45±2.84	6.02±2.96	0.558
**BMI (kg/m^2)**		20.08±2.97	19.32±2.10	20.33±3.18	0.169
**Total Insulin (U/kg/die)**		0.88±0.25	0.89±0.27	0.88±0.24	0.848
**Basal Insulin (U/kg/die)**		0.43±0.15	0.47±0.17	0.42±0.14	0.185
**Prandial Insulin (U/kg/die)**		0.40 [0.29-0.56]	0.35 [0.28-0.51]	0.41 [0.32-0.56]	0.539
**Mean glycemia (sensor)**		172.1±30.0	142.1±10.7	182.7±27.2	**<0.001**
**Blood agar, n=82 (CFU × 10^8/mL)**		10.5±44.4	7.36±5.42	11.6±51.6	0.709
**Sabouraud agar, n=81 (CFU × 10^4/mL)**		0.36±1.61	0.57±2.29	0.30±1.34	0.520
**Mannitol Salt agar, n=82 (CFU × 10^8/mL)**		0.11±0.90	0.001±0.02	0.15±1.05	0.509
**Mitis Salivarius agar, n=82 (CFU × 10^7/mL)**		10.1±21.6	14.9±35.3	8.38±14.1	0.226
**MSB, n=82 (CFU × 10^8/mL)**		0.18±0.34	0.18±0.34	0.13±0.33	0.574
***Veillonella* spp, n(%)**		81 (93.1)	19 (86.4)	62 (95.4)	0.149
***Actinomyces* spp, n(%)**		87 (100)	22 (100)	65 (100)	1
***Actinomyces naeslundii,* n(%)**		41 (47.1)	10 (45.5)	31 (47.7)	0.856
***Treponema denticola*, n(%)**		32 (36.8)	11 (50.0)	21 (32.3)	0.137
***A. actinomycetemcomitans*, n(%)**		87 (100)	22 (100)	65 (100)	1
***Prevotella intermedia*, n(%)**		87 (100)	22 (100)	65 (100)	1
***Porphyromonas gingivalis*, n(%)**		6 (6.9)	3 (13.6)	3 (4.6)	0.149
***Tannerella forsythia*, n(%)**		29 (33.3)	7 (31.8)	22 (33.8)	0.862
***Streptococcus mutans,* n(%)**		43 (49.4)	6 (27.3)	37 (56.9)	**0.016**
***Lactobacillus* spp, n(%)**		87 (100)	22 (100)	65 (100)	1
***Veillonella* spp+ *Streptococcus mutans.*, n(%)**		41 (47.13)	5 (22.7)	36 (55.4)	**0.008**
***T. forsythia + T. denticola + P. gingivalis* (at least 2 of them), n(%)**		16 (18.4)	5 (22.7)	11 (16.9)	0.375

Sample sire, n=87, unless otherwise indicated. Data are expressed as means ± SD, medians and interquartile range [IQR] or proportions. Differences between the two groups of individuals were tested by the unpaired Student's for normally distributed variables, the Mann–Whitney U-test for non-normally distributed variables or the chi-squared test for categorical variables, respectively. *Abbreviations*: BMI, Body Mass Index; CFU, Colony Forming Unit; MSB, Mitis Salivarius sucrose Bacitracin.

Supplementary Table S4. Main clinical, biochemical and microbial characteristic of children and adolescents with type 1 diabetes according to percentage of time above range (TAR, cut-off=25%)

		All (n=87)	TAR<25% (n=19)	TAR>25% (n=68)	p value
Gender	Female (%)	33 (37.9)	6 (31.6)	27 (39.7)	0.519
	Male (%)	54 (62.1)	13 (68.4)	41 (60.3)	
Age (years)		12.54±2.17	13.06±2.33	12.39±2.12	0.233
Diabetes duration (years)		6.13±2.92	6.22±2.85	6.11±2.96	0.881
BMI (kg/m²)		20.08±2.97	19.09±2.11	20.35±3.12	0.100
Total Insulin (U/kg/die)		0.88±0.25	0.87±0.28	0.88±0.24	0.902
Basal Insulin (U/kg/die)		0.43±0.15	0.45±0.18	0.42±0.14	0.508
Prandial Insulin (U/kg/die)		0.40 [0.29-0.56]	0.36 [0.27-0.50]	0.41 [0.32; 0.57]	0.377
Mean glycemia (sensor)		172.1±30.0	139.9±9.76	181.5±27.2	**<0.001**
Blood agar, n=82 (CFU × 10^8/mL)		10.5±44.4	7.00±5.80	11.5±50.4	0.707
Sabouraud agar, n=81 (CFU × 10^4/mL)		0.36±1.61	0.05±0.15	0.44±1.79	0.391
Mannitol Salt agar, n=82 (CFU × 10^8/mL)		0.11±0.90	0.007±0.02	0.14±1.02	0.552
Mitis Salivarius agar, n=82 (CFU × 10^7/mL)		10.1±21.6	15.9±38.0	8.39±13.8	0.186
MSB, n=82 (CFU × 10^8/mL)		0.18±0.34	0.17±0.36	0.13±0.32	0.674
Veillonella spp, n(%)		81 (93.1)	17 (89.5)	64 (94.1)	0.480
Actinomyces spp, n(%)		87 (100)	19 (100)	68 (100)	1
Actinomyces naeslundii, n(%)		41 (47.1)	7 (36.8)	34 (50.0)	0.310
Treponema denticola, n(%)		32 (36.8)	9 (47.4)	23 (33.8)	0.279
A. actinomycetemcomitans, n(%)		87 (100)	19 (100)	68 (100)	1
Prevotella intermedia, n(%)		87 (100)	19 (100)	68 (100)	1
Porphyromonas gingivalis, n(%)		6 (6.9)	3 (15.8)	3 (4.4)	0.084
Tannerella forsythia, n(%)		29 (33.3)	7 (36.8)	22 (32.4)	0.714
Streptococcus mutans, n(%)		43 (49.4)	5 (26.4)	38 (55.9)	**0.023**
Lactobacillus spp, n(%)		87 (100)	22 (100)	65 (100)	1
Veillonella spp+ *Streptococcus mutans.*, n(%)		41 (47.1)	4 (21.0)	37 (54.4)	**0.010**
T. forsythia + T. denticola + P. gingivalis (at least 2 of them), n(%)		16 (18.4)	5 (26.3)	11 (16.2)	0.244

Sample sire, n=87, unless otherwise indicated. Data are expressed as means ± SD, medians and interquartile range [IQR] or proportions. Differences between the two groups of individuals were tested by the unpaired Student's for normally distributed variables, the Mann–Whitney U-test for non-normally distributed variables or the chi-squared test for categorical variables, respectively. *Abbreviations*: BMI, Body Mass Index; CFU, Colony Forming Unit; MSB, Mitis Salivarius sucrose Bacitracin.
